# Case Report: Vogt–Koyanagi–Harada syndrome complicated by secondary glaucoma: diagnostic insights and mechanistic correlations in uveitic ocular hypertension

**DOI:** 10.3389/fmed.2026.1755451

**Published:** 2026-02-10

**Authors:** Fenglong Li, Dongshen Wu, Xiaowei Zhu

**Affiliations:** 1 Department of Ophthalmology, Zhongshan City People’s Hospital, The Affiliated Zhongshan Hospital of Sun Yat-sen University, Zhongshan, China; 2 Faculty of Medicine, Shenzhen University, Shenzhen, China

**Keywords:** case report, ciliary body detachment, secondary angle-closure glaucoma, ultrasound biomicroscopy, Vogt–Koyanagi–Harada syndrome

## Abstract

**Introduction:**

This case highlights an uncommon presentation of Vogt–Koyanagi–Harada (VKH) syndrome, in which acute angle-closure glaucoma (AACG) served as the initial ocular manifestation. This atypical onset may lead to misdiagnosis as primary angle-closure glaucoma. This report adds to the existing literature by emphasizing the diagnostic value of ciliary body imaging and the mechanistic link between inflammatory ciliary body detachment and secondary angle closure.

**Case presentation:**

A 27-year-old woman presented with acute ocular pain, vision loss, and markedly elevated intraocular pressure (IOP). Important clinical findings included conjunctival congestion, corneal edema, medium-depth anterior chambers, and the absence of keratic precipitates or aqueous flare. Ultrasound biomicroscopy revealed a ciliary body detachment, whereas optical coherence tomography revealed multiple serous retinal detachments. The patient was initially misdiagnosed with glaucoma before a revised diagnosis of VKH syndrome-associated secondary AACG was made.

**Interventions and outcomes:**

The patient was treated with intravenous methylprednisolone pulse therapy combined with topical corticosteroids, mydriatic agents, and IOP-lowering agents. Visual acuity and IOP gradually improved, and subretinal fluid completely resolved during follow-up.

**Conclusion:**

VKH syndrome rarely presents with AACG as the initial manifestation. Awareness of this presentation and careful imaging evaluation are essential to avoid misdiagnosis. Early and aggressive anti-inflammatory therapy remains key to reversing both angle closure and retinal pathology. This case highlights the importance of considering autoimmune uveitis in patients with atypical angle-closure glaucoma.

## 1 Introduction

Vogt–Koyanagi–Harada (VKH) syndrome is an autoimmune disease that primarily affects melanocytes and predominantly affects individuals aged 20–50 years. The hallmark of this syndrome is bilateral granulomatous panuveitis, often accompanied by systemic manifestations, including decreased hearing and vision, headache, tinnitus, and vitiligo (1, 2). The course of VKH syndrome is complex, with a high rate of misdiagnosis and missed diagnosis in the early stages. This disease is easily misdiagnosed as sympathetic ophthalmia, birdshot retinochoroidopathy, and Behçet’s disease (3). Moreover, complications are commonly mistaken for primary disease (4). In some cases, VKH syndrome can be misdiagnosed as primary acute angle-closure glaucoma (AACG), presenting with peripheral anterior synechiae and increased intraocular pressure (IOP). However, AACG, as the first ocular manifestation of VKH syndrome, is uncommon (1). Its pathogenesis may involve acute inflammatory edema, disruption of the blood–aqueous humor barrier in the ciliary body, or forward rotation of the iris–lens diaphragm (5, 6). Herein, we present a case of VKH syndrome with AACG as the initial manifestation and investigate its underlying pathogenesis.

## 2 Case presentation

**Figure 1:**
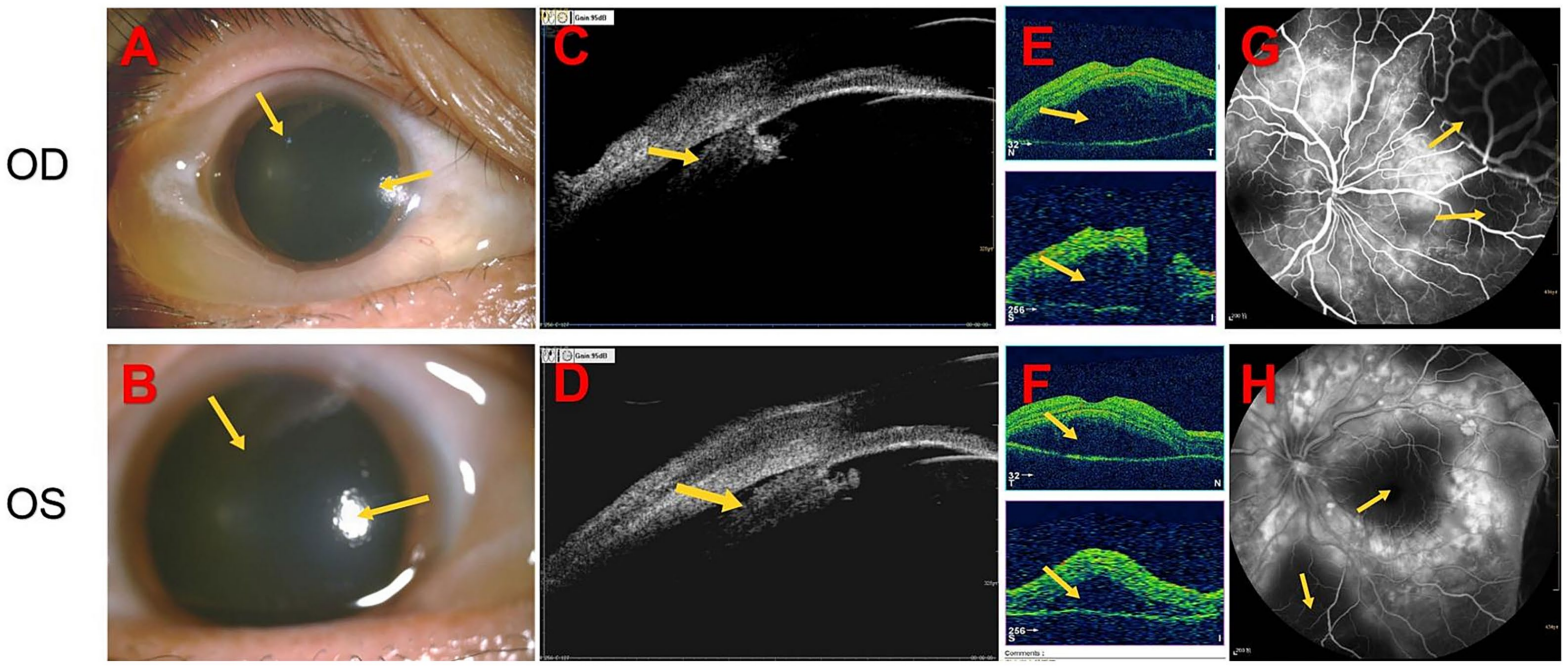
After outpatient pupil dilation, anterior segment photography shows conjunctival congestion with edema, corneal opacity, absence of keratic precipitates, medium-deep anterior chambers, and absence of aqueous flare **(A,B)**. Ultrasound biomicroscopy reveals ciliary body detachment **(C,D)**. Optical coherence tomography demonstrates multiple serous retinal detachments involving the posterior pole in both eyes **(E,F)**. Fundus fluorescein angiography shows diffuse fluorescein leakage and staining **(G,H)**.

**Figure 2:**
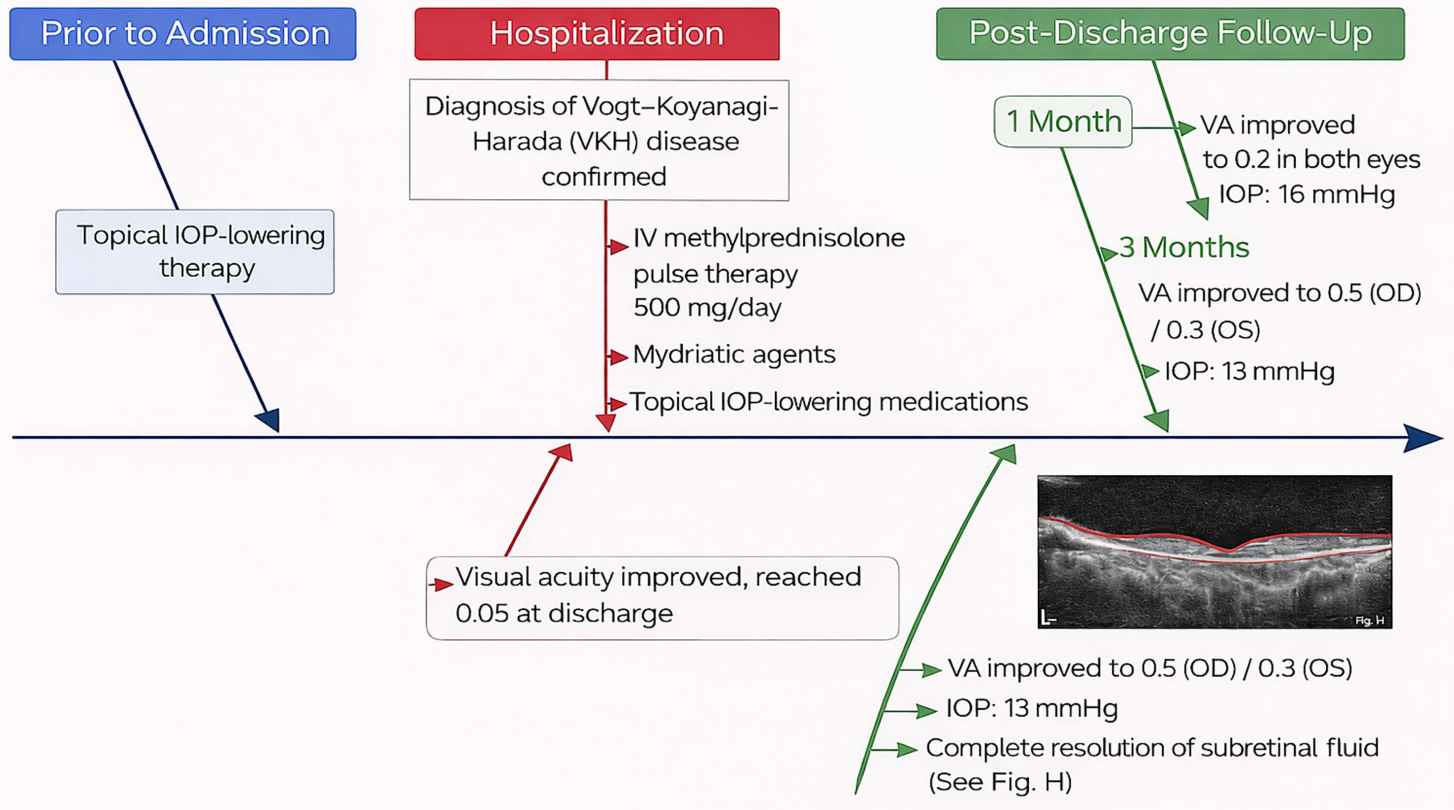
Diagnostic and therapeutic course of the patient from pre-admission to follow-up, illustrating the sequence of management and disease progression. IOP, intraocular pressure.

A 27-year-old woman was admitted for treatment of red eyes, ocular pain, increased IOP, and self-perceived decreased vision (near-absent light perception) for 2 days. The patient had been diagnosed with glaucoma at another hospital 1 day before admission and underwent glaucoma treatment, after which IOP decreased; however, vision continued to deteriorate progressively. After admission, relevant examinations were performed, and the findings suggested that the initial manifestation was VKH syndrome with AACG. Anterior segment photography (after pupil dilation) showed conjunctival congestion with edema, corneal opacity, absence of keratic precipitates, medium-depth anterior chambers, and absence of aqueous flare (−) (Figures 1A,B). Notably, ultrasound biomicroscopy revealed ciliary body detachment (Figures 1C,D). Secondary angle-closure glaucoma in eyes with uveitis can result from several mechanisms, including angle closure with pupillary block, which occurs when anterior chamber inflammation leads to 360° posterior synechiae, as well as inflammation and edema causing forward rotation of the ciliary body and angle closure. Optical coherence tomography of both eyes revealed multiple serous retinal detachments at the posterior pole (Figures 1E,F). Fundus fluorescein angiography revealed diffuse fluorescein leakage and staining (Figures 1G,H). Before admission, the patient had received topical IOP-lowering therapy. After admission, following the confirmation of a diagnosis of VKH syndrome, intravenous methylprednisolone pulse therapy (500 mg/day) was initiated in combination with mydriatic agents and topical IOP-lowering medications. Visual acuity gradually improved and reached 0.05 at discharge. At the 1-month follow-up, visual acuity had improved to 0.2 in both eyes, with an IOP of 16 mmHg. At the 3-month follow-up, visual acuity further improved to 0.5 in the right eye and 0.3 in the left eye, with an IOP of 13 mmHg, and complete resolution of subretinal fluid was observed. The patient’s diagnostic and therapeutic course is shown in Figure 2.

## 3 Diagnosis of VKH syndrome

The diagnosis of VKH syndrome was established according to the revised diagnostic criteria of the International Committee on VKH Disease. This patient exhibited the following features:

Bilateral ocular involvement.
Serous retinal detachments on optical coherence tomography.
Ciliary body detachment, suggesting choroidal inflammation.
Systemic features, including headache.
Absence of keratic precipitates, infectious symptoms, or a history of trauma.

HLA-DR4/DRB104 testing, while supportive, was not performed because the diagnosis was sufficiently supported by clinical findings and multimodal imaging.

## 4 Discussion

### 4.1 Pathophysiological interplay

The exact pathophysiological mechanisms underlying the association between VKH syndrome and AACG remain incompletely understood (7, 8). Current evidence suggests that the primary mechanisms include posterior iris adhesion with pupillary block, anterior chamber angle occlusion, trabecular meshwork inflammation, accumulation of inflammatory cells near the trabecular meshwork, and long-term use of glucocorticoids (9). Notably, this patient did not exhibit angle recession but instead demonstrated ciliary body detachment, which we believe was the primary cause of elevated IOP. This finding is consistent with that of previous reports (10). Detachment of the ciliary body leads to zonular laxity, allowing anterior displacement of the lens and subsequent compression of the iris, resulting in pupillary block. This obstruction impairs aqueous humor flow from the posterior to the anterior chamber, ultimately leading to the development of malignant glaucoma. Differential diagnoses, including sympathetic ophthalmia, infectious uveitis, central serous chorioretinopathy, and primary AACG, were excluded based on clinical features and imaging findings.

### 4.2 Treatment considerations

Management of VKH syndrome-associated AACG requires a comprehensive approach that addresses both the underlying inflammatory and mechanical components of the disease (11, 12). In this case, the patient underwent the following interventions.

### 4.3 Systemic corticosteroids

Systemic corticosteroids (intravenous methylprednisolone, 500 mg/day for 5 days) were administered to treat the acute inflammatory process. This high-dose therapy aims to reduce inflammation, alleviate pupillary block, and prevent further complications, such as retinal detachment. The dosage was gradually tapered over several weeks to minimize adverse effects associated with prolonged steroid use, in accordance with standard management strategies for autoimmune uveitis (13, 14).

### 4.4 Topical corticosteroids

Topical corticosteroid eye drops were administered to reduce anterior segment inflammation. The dosage and frequency were adjusted according to the severity of anterior chamber inflammation and corneal edema and were gradually tapered once inflammation was adequately controlled.

### 4.5 Mydriatic therapy

Mydriatic agents were used to induce pupil dilation and relieve pupillary block caused by anterior displacement of the lens. Mydriatic therapy helped maintain pupillary dilation and reduce elevated IOP secondary to forward displacement of the lens–iris diaphragm resulting from ciliary body detachment.

### 4.6 Changes in interventions

During the course of treatment, the initial priority was controlling elevated IOP, a critical concern in secondary angle-closure glaucoma. As the patient’s condition improved, corticosteroid therapy was gradually tapered to reduce potential adverse effects. Visual acuity and IOP normalized, and subretinal fluid progressively resolved during follow-up visits at 1 and 3 months.

### 4.7 Rationale for treatment changes

The initial use of systemic corticosteroids was essential for controlling inflammation and secondary angle closure caused by ciliary body detachment and pupillary block.

Corticosteroids were tapered as the patient’s condition stabilized, following established protocols to minimize complications associated with prolonged steroid use while maintaining inflammatory control.

The use of mydriatic agents was critical for preventing further IOP elevation and improving patient comfort by alleviating pupillary block.

These treatment strategies are consistent with established clinical guidelines for VKH syndrome-associated AACG and were selected to address both the acute and chronic aspects of the disease while accounting for the patient’s individual response (7, 14, 15).

## 5 Conclusion

This case report provides important insights into the pathogenesis and clinical decision-making involved in VKH syndrome-associated AACG. Early recognition of systemic symptoms, appropriate imaging evaluation, and a multidisciplinary treatment approach are essential for optimizing outcomes and preventing irreversible visual loss.

## Data Availability

The original contributions presented in the study are included in the article/supplementary material, further inquiries can be directed to the corresponding author.
